# Multi-Omics Advances in Major Depressive Disorder for Molecular Insights, Biomarker Discovery, and Therapeutic Development

**DOI:** 10.14336/AD.2025.1075

**Published:** 2026-10-15

**Authors:** Abdelbaset Mohamed Elasbali, Mohd Adnan, Ahmed S. Ali, Anas Shamsi, Md. Imtaiyaz Hassan

**Affiliations:** ^1^Department of Clinical Laboratory Science, College of Applied Medical Sciences-Qurayyat, Jouf University, Sakakah, Saudi Arabia.; ^2^King Salman Center for Disability Research, Riyadh 11614, Saudi Arabia.; ^3^Department of Biology, College of Science, University of Ha'il, Ha'il, Saudi Arabia.; ^4^Department of Physical Therapy and Health Rehabilitation, College of Applied Medical Sciences, Jouf University, Al-Qurayyat, Saudi Arabia.; ^5^Centre of Medical and Bio-Allied Health Sciences Research, Ajman University, Ajman, United Arab Emirates.; ^6^Centre for Interdisciplinary Research in Basic Sciences, Jamia Millia Islamia, Jamia Nagar, New Delhi 110025, India.

**Keywords:** Major depressive disorder, multiomics, genomics, transcriptomics, proteomics, epigenomics, metabolomics

## Abstract

Major depressive disorder (MDD) affects hundreds of millions worldwide and remains a major unmet clinical need because conventional monoaminergic agents achieve remission in fewer than half of patients and leave a substantial treatment-resistant subgroup. To address this gap, integrative multi-omics approaches, combined with computational systems biology, are being used to dissect its multifactorial etiology. Here, we review key findings from large Genome-wide association studies (GWAS), single-cell and spatial transcriptomics, proteomics, and metabolomics that converge on disrupted neuroplasticity, immune-inflammatory signaling, HPA-axis dysregulation, mitochondrial metabolism, and gut-brain interactions. We describe how AI-driven network modeling and structure-based drug design (SBDD) are translating multi-omics signals into candidate biomarkers and mechanism-based therapeutics, for example, N-methyl-D-aspartate/glutamatergic modulators, kappa opioid antagonists, anti-inflammatory agents, and epigenetic modulators. We highlighted the clinical implications, specifically molecularly stratified biomarkers for patient selection, trial enrichment, and structure-guided optimization of rapid-acting antidepressants and microbiome-based interventions. Finally, we discuss the limitations and immediate translational priorities that emphasize the trajectory from multi-omics discovery to precision psychiatry for MDD.

## Introduction

Major depressive disorder (MDD) is a leading cause of global disability and affects a large and growing portion of the world population. Recent global burden analyses estimate >300 million people currently live with depressive disorders, and prevalence and disability have risen substantially over the last decade. The health, social, and economic impacts of MDD, including higher morbidity, reduced productivity, and elevated suicide risk, underscore the urgent need for improved diagnostics and mechanism-based therapies. MDD is defined by chronic low mood, anhedonia, and cognitive and physical impairment [1, 2].

It is characterized by complex neurobiological dysfunction in interacting brain areas, including the prefrontal cortex, amygdala, and hippocampus. These dysfunctions induce imbalances in neurotransmitter systems such as serotonin, norepinephrine, and dopamine, as well as disruption of essential signaling systems like the hypothalamic-pituitary-adrenal (HPA) axis, inflammatory cytokine cascades, and neurotrophic factor signaling [3-5]. The complexity of the etiology of MDD, encompassing genetic factors, environmental stressors, and epigenetic changes, accounts for the inadequacy of the conventional pharmacotherapies like selective serotonin reuptake inhibitors (SSRIs) and serotonin-norepinephrine reuptake inhibitors (SNRIs) [6, 7]. These monoamine-targeted treatments induce remission in less than half of the patients, resulting in a substantial number of patients with treatment-resistant depression (TRD) [8]. The inefficacy of current treatments provides the rationale for exploring novel paradigms to demystify the molecular mechanisms underlying MDD and to identify new targets for more effective and targeted therapies.

The emergence of multi-omics technologies comprising genomics, transcriptomics, proteomics, metabolomics, and epigenomics has transformed the dissection of complex neuropsychiatric disorders like MDD [9-11]. By simultaneously probing different layers of biology, multi-omics approaches provide a holistic picture of the molecular landscape, unveiling complex networks of gene regulation, protein-protein interactions, and metabolic pathways modulated by MDD [12-14]. A recent review focusing on convergent molecular pathways highlighted pathways such as TGF-β1 signaling as a cross-disease mediator between depression and neurodegeneration; integrating such pathway-level insights complements omics-driven target discovery and suggests shared therapeutic mechanisms across disorders. We briefly compare and extend those observations by explicitly connecting pathway signatures to SBDD opportunities and clinical trial enrichment strategies [15].

Genomic studies, especially large-scale genome-wide association studies (GWAS), have implicated genetic risk factors and single-nucleotide polymorphisms (SNPs) of MDD, which target pathways in neurotransmitter signaling, synaptic plasticity, neuroinflammation, and HPA axis regulation [16]. Salient genes, like *SLC6A4* (encoding the serotonin transporter), *BDNF* (brain-derived neurotrophic factor), and *FKBP5* (regulating glucocorticoid receptors), indicate a heritable component of MDD; however, they explain only a percentage of the disorder, emphasizing the contribution of other regulatory mechanisms [17, 18]. Transcriptomic studies, including RNA sequencing and single-cell transcriptomics, have shown dysregulated networks of gene expression, which modulate synaptic function, immune processes, and neuroplasticity, and also indicate a global transcriptional reprogramming in MDD [19-21]. Epigenetic modifications like DNA methylation, histone modifications, and regulation of non-coding RNAs further regulate gene expression in response to environmental cues, which modulate the onset and progression of the disorder [13, 22, 23].

These findings have been complemented by proteomic and metabolomic investigations that have recognized biochemical changes important to MDD pathophysiology. Proteomics has shown disturbed synaptic proteins, immune-related compounds, and signaling cascades, whereas metabolomics has revealed derangements in energy metabolism, neurotransmitter biosynthesis, and lipid signaling [24]. The integration of these multi-omics datasets with systems biology and artificial intelligence (AI) approaches allows the building of dynamic molecular models, which make it possible to identify biomarkers for diagnosis, prognosis, and response to treatment, as well as new therapeutic targets [25, 26]. Such progress sets the stage for precision psychiatry, in which patient stratification by molecular subtypes can maximize treatment responses. Emerging treatments, such as neurostimulation, gene editing, and small-molecule inhibitors against specific pathways, demonstrate the translational value of multi-omics in meeting the unmet needs of MDD patients [27, 28].

In proteomics, structure-based drug discovery (SBDD), relying on high-resolution three-dimensional structural data to design specific treatments for MDD, is at the heart of this new paradigm [29]. SBDD enables the precise targeting of important proteins and molecular complexes implicated in MDD through the integration of different biological data with structural data from techniques such as X-ray crystallography [30], cryo-electron microscopy, and molecular dynamics simulations [31, 32]. For example, structural elucidation of neurotransmitter receptors, HPA axis regulators, and neuroinflammatory mediators enables the design of small-molecule inhibitors with increased potency and selectivity. This strategy not only speeds the discovery of new drug candidates but also reduces off-target effects, overcoming the flaws of conventional antidepressants [33, 34]. As the molecular design of MDD-targets is further mapped, SBDD promises revolutionary potential for the formulation of next-generation antidepressants that are more efficacious and have fewer side effects.

This review aims to synthesize recent multi-omics discoveries (2020-2025) and to frame them within translational pipelines that connect molecular signatures to therapeutic development. Unlike prior overviews that treated omics layers separately, we (1) integrate multi-modal molecular evidence with structure-based drug design (SBDD), (2) evaluate evidence strength for emerging therapeutic classes (rapid-acting glutamatergic agents, kappa opioid antagonists, anti-inflammatories, psychedelics, and microbiome interventions), and (3) map ongoing clinical trials to molecular subtypes to highlight immediate paths for biomarker-guided trials [35]. We conclude with prioritized recommendations to accelerate clinical translation and address methodological gaps. The combination of SBDD with multi-omics data represents an exciting future direction in MDD therapeutics, holding promise for more efficient and personalized treatments targeting the complex neurobiological substrates of this debilitating illness. An overview of this integrative multi-omics strategy and its relevance to precision medicine in MDD is illustrated in Figure 1.

**Figure 1.**
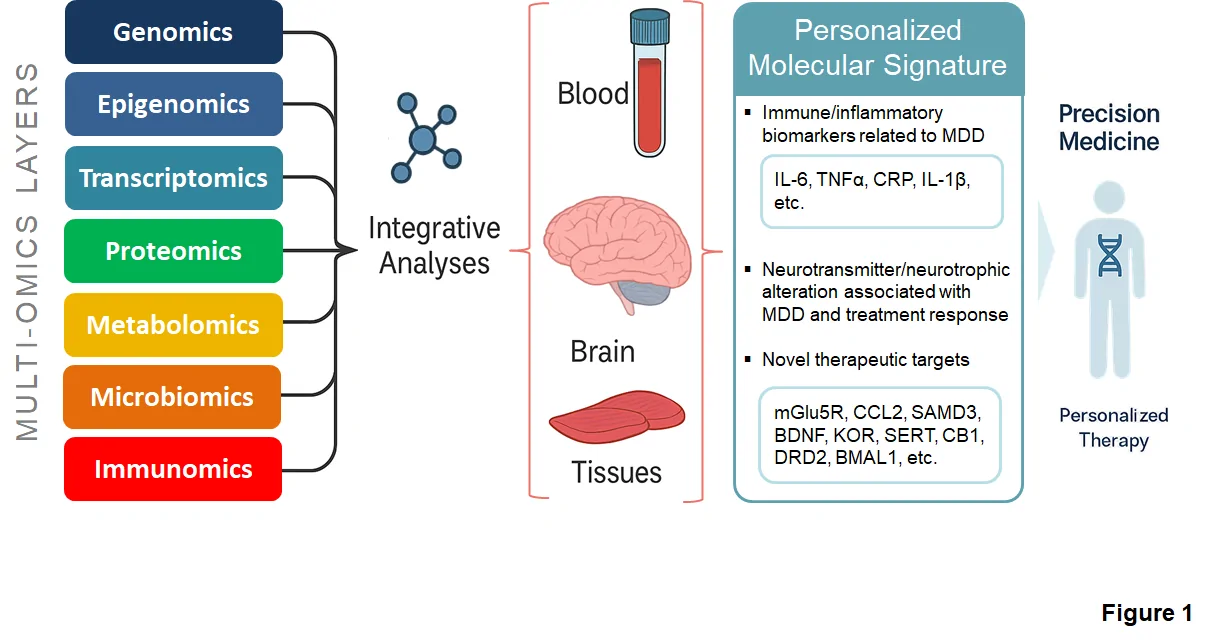
**Integrative multi-omics framework for precision medicine development**. The figure illustrates a comprehensive approach combining multiple omics layers, genomics, epigenomics, transcriptomics, proteomics, metabolomics, microbiomics, and immunomics, for integrative analyses. Data derived from various biological sources, such as blood, brain, and other tissues, are analyzed collectively to identify a personalized molecular signature. This integrative molecular profiling enables the design of precision medicine strategies and the implementation of personalized therapy, allowing for targeted interventions tailored to an individual’s unique molecular and physiological landscape.

## Multi-omics approaches in MDD

Multi-omics approaches, which integrate data from genomics, transcriptomics, proteomics, and metabolomics, offer a comprehensive framework for unraveling the molecular underpinnings of MDD. Among these, genomics has seen remarkable advancements, revolutionizing our understanding of MDD and opening new therapeutic opportunities [36]. The details below focus on how genomic advancements enhance MDD therapeutics, highlighting key methodologies, findings, and their implications.

## Genomics

Genomic studies have significantly expanded our understanding of MDD by revealing the complex genetic architecture underlying its susceptibility and response to treatment. Genome-wide association (GWAS) studies, such as those conducted by the Psychiatric Genomics Consortium, have identified numerous risk loci; however, single variants rarely reach genome-wide significance due to the polygenic nature of MDD [37, 38]. Key findings include correlations with genes such as SAMD3 and GRIN2B, which are associated with the age of onset and may have protective or risk-modifying effects [39]. Next-generation sequencing technologies, including whole-genome sequencing (WGS) [40] and whole-exome sequencing (WES), have also facilitated the identification of single-nucleotide polymorphisms (SNPs), copy number variations (CNVs), and rare variants that underlie MDD risk [41]. These initiatives have brought genes that mediate synaptic plasticity, neurodevelopment, and neurotransmitter signaling into focus, such as DRD2 (dopamine receptor D2) and SLC6A4 (serotonin transporter) [42]. Furthermore, studies in pharmaco-genomics have determined potential SNPs that could forecast antidepressant treatment response, enabling tailored treatment strategies (Table 1).

This genomic model has provided important insights into the biological mechanisms underlying MDD, setting the stage for precision medicine [43]. Recent large-scale GWAS and fine-mapping efforts have expanded the repertoire of MDD-associated loci. High-confidence genes include SAMD3, GRIN2B (GluN2B), NEGR1, BDNF, and loci linked to synaptic function and neuronal development. Polygenic risk scores (PRS) now capture cumulative risk across hundreds of common variants and show modest predictive value for disease onset and some treatment responses; however, PRS performance improves when integrated with clinical and environmental data. Recent multi-ancestry GWAS have increased locus discovery and reduced Eurocentric bias, enabling better gene prioritization and causal inference for targets implicated in synaptic plasticity and glutamatergic signaling [15].

**Table 1:** **Multi-omics findings in MDD**. Summary of representative genomic, transcriptomic, proteomic, and metabolomic alterations reported in major depressive disorder^*^.

Genomics approaches	Description	Mechanisms Investigated	Example Studies	Ref.
**Genome-Wide Association Studies (GWAS)**	Tests millions of single-nucleotide polymorphisms (SNPs) across the genome to identify variants associated with MDD.	Genetic variants contributing to MDD risk include those affecting neurotransmitter systems, synaptic plasticity, and stress response.	Identified 697 SNPs and 308 genes, implicating neuronal differentiation and receptor clustering (e.g., DRD2, CYP7B1).	[60]
**Transcriptomics**	Analyzes gene expression profiles in brain tissue or peripheral samples to identify differentially expressed genes (DEGs).	Dysregulation of brain transcriptome, inflammatory pathways, apoptosis, and immune-related pathways.	Postmortem brain studies show dysregulation of synaptic and immune-related genes; North Indian study identified DEGs linked to inflammation and treatment response.	[61]
**Phenome-Wide Association Studies (PheWAS)**	Explores phenotypic consequences of genetic variants using large-scale biobanks and electronic health records.	Pleiotropic effects of MDD-associated genes on comorbid conditions (e.g., insomnia, anxiety).	PheWAS linked MDD variants to phenotypes like insomnia, revealing shared genetic liabilities.	[62]
**Mendelian Randomization**	Uses genetic variants as instrumental variables to infer causal relationships between MDD and other traits.	Causal pathways, e.g., between MDD and body mass index or insomnia.	Showed directional association between BMI and MDD risk, implicating modifiable environmental factors.	[63]
**Expression Quantitative Trait Loci (eQTL) Mapping**	Maps SNPs to gene expression levels to understand functional impacts of genetic variants.	Gene expression regulation in brain regions (e.g., prefrontal cortex, cerebellum) affecting neuronal function.	Enriched MDD/insomnia gene expression in glutamatergic and GABAergic neurons in cerebellum and cortex.	[64]
**Copy Number Variation (CNV) Analysis**	Identifies large structural DNA variations (e.g., deletions, duplications) associated with MDD.	Rare variants affecting neurodevelopmental pathways and synaptic function.	UK Biobank study found CNVs (e.g., 1q21.1, 16p11.2 duplications) linked to MDD and neurodevelopmental disorders.	[65]
**Whole-Exome/Genome Sequencing (WES/WGS)**	Detects rare and low-frequency sequence variants across coding (WES) or entire genome (WGS).	Rare variants in genes like SIRT1 and LHPP affecting stress response and synaptic signaling.	CONVERGE study identified variants near SIRT1 and LHPP in Chinese women with recurrent MDD.	[66]
**Network and Pathway Analysis**	Integrates genetic and transcriptomic data to construct disease-specific molecular networks.	Functional interactions of genes in pathways like neuroactive ligand-receptor interaction, dopaminergic synapse.	Identified 143 MDD-related genes and pathways like serotonin and dopamine signaling using systems biology approaches.	[67]
**Epigenetics (DNA Methylation Studies)**	Examines chemical modifications to DNA that affect gene expression without altering sequence.	Epigenetic regulation of stress response and inflammation genes.	DNA methylation patterns linked to oxidative stress and HPA axis dysfunction in MDD.	[68]
**Polygenic Risk Scores (PRS)**	Aggregates effects of multiple genetic variants to predict MDD risk or treatment response.	Cumulative genetic liability influencing MDD susceptibility and treatment outcomes.	PRS explained 5.7% of MD variance in European samples; used for risk prediction and therapy stratification.	[69]

* Data is compiled from primary peer-reviewed studies listed in the References column.

Recent technological innovations in genomic research have significantly enhanced the utility of these technologies in MDD studies and therapeutics. High-throughput NGS platforms have reduced costs and accelerated sequencing, enabling large-scale studies across multiple populations [44]. This has remedied the historical limitations of GWAS, typically constrained to European samples, by incorporating underrepresented populations to reveal population-specific variants [45]. The second achievement is the inclusion of polygenic risk scores (PRS), which summarize the combined effect of many genetic variants to estimate an individual's risk of developing MDD or predicting treatment response [46]. PRS models have been promising to stratify individuals into groups for tailored treatment regimens, for example, to identify those who will respond to selective serotonin reuptake inhibitors (SSRIs) [47]. Moreover, epigenomics advances, which observe heritable alterations in gene expression without altering the DNA sequence, have elucidated how environmental conditions, including stress or trauma, affect gene regulation in MDD [17, 48]. Methods such as chromatin immunoprecipitation sequencing (ChIP-seq) and DNA methylation profiling have identified epigenetic markers that may serve as therapeutic targets [49].

## Transcriptomics

Transcriptomics, which analyzes the whole complement of RNA transcripts [50] produced from the genome, has been an important tool for identifying the molecular underpinnings of MDD [51]. Transcriptomics provides a snapshot at a given time point of gene expression and how the genetic information is being translated into functional molecules [52]. The recent advances in transcriptomics, including single-cell RNA sequencing (scRNA-seq) and spatial transcriptomics, have significantly improved our understanding of MDD by allowing cell-type-specific analysis [53]. For instance, scRNA-seq has defined cell-type-specific transcriptional profiles of neurons, astrocytes, and microglia and emphasized cell-specific mechanisms in disease pathology [54]. Experiments have reported dysregulated expression of genes encoding synaptic plasticity, neuroinflammation, and stress response, such as BDNF and FKBP5, in critical brain regions such as the prefrontal cortex, hippocampus, and amygdala, all of which are implicated in mood regulation and cognitive function [55]. Transcriptomic profiling of postmortem brain and peripheral blood has also identified biomarkers such as IL-1RA and CRP and emphasized the involvement of immune pathways in MDD. These reports provide a strong foundation for the identification of novel therapeutic targets and the generation of precision therapies for MDD, transforming our management of the complex psychiatric disorder [56].

Advances in transcriptomics technologies have further enhanced their utility in MDD research. Spatial transcriptomics, which localizes gene expression in the spatial architecture of tissue, has provided spatially resolved data on MDD pathology [57]. By preserving cellular spatial organization, the method has elucidated region-specific changes in gene expression in the brain, including those affecting neuroinflammatory pathways in the hippocampus [58]. These findings underscore the importance of spatial context in MDD research and the development of region-specific therapeutic targets.

Yet another breakthrough is the integration of transcriptomics with the other omics. Multi-omics strategies integrate transcriptomic profiles with genomic, epigenomic, and proteomic information to build end-to-end molecular networks. For instance, the integration of transcriptomics with epigenomics has uncovered how histone modifications and DNA methylation control gene expression in MDD, defining new regulatory pathways [59]. Likewise, the integration of transcriptomics with metabolomics has been linked to dysregulated gene expression and aberrant neurotransmitter metabolism, such as the serotonin and glutamate pathways, which are crucial for mood regulation. These integrative analyses facilitate our capacity to detect biomarkers and therapeutic targets that signify the interaction of multiple biological layers [38].

## Proteomics

Mass spectrometry (MS)-based proteomics enables the high-throughput investigation of protein expression, post-translational modifications, and interactions that inform the molecular landscape of MDD (Table 2). Biofluid (e.g., plasma, cerebrospinal fluid) and brain tissue proteomics identify dysregulated proteins and pathways beyond monoamine systems [70]. Proteomic investigations uniformly indicate immune-inflammatory dysregulation in MDD, with increased plasma levels of interleukin-1 receptor antagonist (IL-1RA) and C-reactive protein (CRP) correlating with MDD subgroups, e.g., TRD [71]. These observations are directed towards anti-inflammatory treatment, e.g., infliximab, a tumor necrosis factor-alpha (TNF-α) inhibitor, which is effective in MDD patients with elevated CRP levels [72].

A milestone study by Pan et al. (2018) developed a biomarker panel comprising gamma-aminobutyric acid (GABA), dopamine, tyramine, and kynurenine, demonstrating high diagnostic accuracy (AUC > 0.85) for late-life depression [73]. Dysregulation of the kynurenine pathway, induced by indoleamine 2,3-dioxygenase (IDO) activation, produces neurotoxic metabolites, with IDO inhibitors displaying preclinical potential [74]. Proteomic profiling also detects disruptions in synaptic plasticity and oxidative stress pathways, with decreased brain-derived neurotrophic factor (BDNF) and TrkB levels driving BDNF-modulating treatments, such as ketamine [75]. Oxidative stress markers, such as superoxide dismutase, show potential for antioxidant-based treatments [76].

**Table 2:** **Overview of omics technologies**. Comparison of platforms used in MDD research, including input material, coverage, strengths, and limitations^*^.

Approaches	Description	Mechanisms Investigated	Example Studies	Ref.
**Proteomics: Serum/Plasma Proteomic Profiling**	Uses mass spectrometry (e.g., MRM-MS) to quantify proteins in blood samples, identifying differentially expressed proteins (DEPs) in MDD patients vs. controls.	Dysregulation in immune response, inflammation, lipid metabolism, and oxidative stress pathways.	Netherlands Study of Depression and Anxiety identified DEPs in current MDD (e.g., inflammatory markers, lipid metabolism proteins), with partial replication in independent cohorts.	[82]
**Proteomics: Cerebrospinal Fluid (CSF) Analysis**	Analyzes CSF proteins to identify biomarkers closer to brain pathophysiology using mass spectrometry.	Neurotransmitter signaling, neurotrophic factors, and immune/inflammatory pathways.	The study consistently altered proteins, such as BDNF and inflammatory mediators, in MDD, indicating their potential for diagnostic use.	[83]
**Proteomics: Brain Tissue Analysis**	Examines postmortem brain tissue (e.g., prefrontal cortex, hippocampus) for protein changes using iTRAQ-based proteomics.	Synaptic transmission, energy metabolism (glycolysis, oxidative phosphorylation), and cytoskeletal changes.	Studies in anterior cingulate cortex showed altered synaptic and energy metabolism proteins; animal models linked DRP-2 to treatment resistance.	[84]
**Proteomics: Protein Interactomics**	Maps protein-protein interactions to identify networks involved in MDD pathology.	Interactions in neurosignaling, immune response, and mitochondrial function.	STRING analysis revealed protein networks in dorsolateral prefrontal cortex linked to synaptic and immune dysregulation.	[85]
**Metabolomics: Untargeted Metabolomics**	Uses MS-based platforms (LC-MS, GC-MS) to profile a broad range of metabolites in plasma, urine, or CSF.	Dysregulated amino acid metabolism, lipid profiles, and energy metabolism (glycolysis, TCA cycle).	Identified altered metabolites in MDD, including monoamine neurotransmitters, amino acids, and lipids; kynurenine and acylcarnitine consistently linked to MDD.	[86]
**Metabolomics: Targeted Metabolomics**	Quantifies specific metabolites (e.g., amino acids, neurotransmitters) using MS-based methods (e.g., HILIC-MS/MS).	Monoamine neurotransmitters, amino acid metabolism (e.g., serine, glycine), and mitochondrial energetics.	Targeted analysis found altered urocanic acid and acylcarnitine in MDD serum, linked to depression severity.	[87]
**Metabolomics: Pseudotargeted Metabolomics**	Combines untargeted and targeted approaches for broader metabolite coverage with improved quantification.	Amino acid metabolism, lipid remodeling, and energy metabolism pathways.	Identified 14 metabolites (e.g., acetylcarnitine) in MDD serum, validated for diagnostic potential; linked to mitochondrial dysfunction.	[88]
**Integrated Proteomics and Metabolomics**	Combines iTRAQ-based proteomics and GC-MS metabolomics to study brain regions (e.g., hippocampus, cerebellum) in animal models or human samples.	Energy metabolism (glycolysis, TCA cycle), amino acid metabolism, and mitochondrial dysfunction.	CUMS rat model studies showed 30 metabolites and 170 proteins altered in hippocampus, linked to energy metabolism and synaptic dysfunction.	[89]
**Pharmacometabolomics**	Maps metabolite changes post-antidepressant treatment to understand drug mechanisms and predict response.	Metabolic changes in response to antidepressants (e.g., SSRIs, TCMs) affecting lipid and amino acid pathways.	Research found that after treatment, there were changes in acylcarnitine and lipid levels, suggesting that improving energy production in mitochondria could be a way to treat depression.	[90]
**Metabolite Set Enrichment Analysis (MSEA)**	Uses databases like KEGG to identify dysregulated metabolic pathways in MDD.	Dysfunction in glycine, serine, arginine, proline, and phenylalanine/tyrosine metabolism.	MSEA revealed altered glycine and serine metabolism in MDD patients, with L-glutamic acid and pyruvic acid linked to depression severity.	[91]

* Reported values represent typical ranges and may vary across platforms; cited references correspond to primary methodological sources.

## Metabolomics

Metabolomics gives a glimpse of metabolic alterations in MDD with disordered profiling pathways in biofluids (plasma, urine) and predictors of treatment response [77]. Amino acids, such as tryptophan, are involved in the pathophysiology of serotonin synthesis reduction, which can be restored by treatment with selective serotonin reuptake inhibitors (SSRIs) such as escitalopram [78]. Fatty acid metabolism is additionally disturbed, with low omega-3 PUFA associated with symptom severity; PUFA supplementation is beneficial as an add-on treatment [79]. Hydroxy sphingomyelins or glycerophospholipids are markers for antidepressant response, where higher levels of sphingomyelin are associated with the efficacy of escitalopram; while glycerophospholipid metabolism is affected by ketamine [80]. Metabolomics enables drug repurposing, as demonstrated in the past (e.g., the diabetes drug metformin has been shown in preclinical studies to have anti-cancer properties). Metformin is a diabetes medication that ameliorates depressive-like behaviors by modulating neuroinflammatory pathways and amino acid metabolism [81]. These findings highlight the role of metabolomics in personalized therapy and therapeutic development (Table 2).

## Epigenomics

Epigenetic modifications, including DNA methylation, histone modifications, and non-coding RNAs, mediate gene-environment interactions in MDD, with profiling in peripheral blood and postmortem brain tissue identifying regulatory mechanisms [92]. Differentially methylated CpG positions (DMPs) in genes like BDNF and NR3C1 correlate with reduced gray matter volume in the prefrontal cortex, contributing to structural brain changes and offering targets for epigenetic therapies like histone deacetylase (HDAC) inhibitors (e.g., valproate) [93]. MicroRNA (miRNA) profiling identifies upregulated miRNAs, such as hsa-mir-589, regulating immune-related pathways, with miRNA-based therapeutics like antagonism under exploration, and hsa-mir-146a predicting SSRI efficacy [94]. Early-life stress induces hypermethylation of the glucocorticoid receptor gene (NR3C1), impairing stress response regulation and increasing MDD risk, with DNA methyltransferase inhibitors under investigation [95]. Multi-omics integration of proteomics, metabolomics, and epigenomics reveals overlapping pathways like neuroinflammation and synaptic regulation, with machine learning enhancing biomarker discovery and treatment outcome prediction, paving the way for precision medicine in MDD [96].

## Multi-Omics Integration

The integration of omics, or multi-omics, is the merging of information from levels of omics, such as genomics and proteomics, to form a complete patient profile for diseases like MDD. Physicians can't see the level of omics as clearly as a picture at the level of an individual gene or protein [10]. And this is why multi-omics is a powerful tool in profiling disorder conditions at the personal level. We can now visualize a kind of picture emerging at the level of MDD with a resolution that was not possible even 10 years ago [97]. At the base of the pathway, it is clear that the omics layers offer real intelligence at the individual level for precision medicine. We can now use omics intelligence to guide us in the potential development of biomarkers [98]. For example, combining GWAS with metabolomics has differentiated early-onset from adult-onset MDD, uncovering unique metabolic signatures such as modified tryptophan metabolism in early-onset instances [99].

Transcriptomics reveals reduced expression of neurotrophic factors in brains affected by MDD. Proteomics complements this by demonstrating increased inflammatory proteins and cytokine-related signaling [100]. Metabolomics elucidates small-molecule changes, such as those in the disrupted pathways of kynurenine. In contrast, epigenomics highlights DNA methylation in stress-sensitive genes and their associated pathways, which tend to either aggravate or ameliorate, thereby regulating them [101]. Machine learning helps analyze data, recognizing features captured in the intricate streams of data. Supervised algorithms, such as random forests, are used to forecast the subtypes of MDD and anticipate their therapeutic responses [102]. Unsupervised clustering exposed previously unknown patient subgroups based on composite multi-omics profiles. With network analysis, maps interactions identified hub genes or proteins believed to be vital in MDD pathways [103]. For example, a 2023 study merged transcriptomics and proteomics, aiming to map immune-inflammation networks and identified interleukin-6 as a principal node of treatment-resistant MDD.

Inconsistent data formats, high dimensionality, and batch effects pose significant challenges to multi-omics integration. Standardized preprocessing, including normalization, imputation, and harmonized metadata, reduces these sources of error and improves reproducibility [19]. Other issues, such as preserving multi-omics interactions while reducing dimensionality, can be addressed using advanced tools like tensor decomposition. Rigorous cross-validation strengthens biomarker identification while preventing overfitting in small sample sizes.

In the case of MDD, multi-omics can significantly alter the disease situation [104]. Diagnostics shift to rely on genetic, proteomic, and metabolomic markers instead of symptom-based ones, increasing accuracy with thresholds surpassing the 95% mark. For another example, a 2024 study reported a multi-omics predictor with ~85% accuracy in predicting MDD relapse [105]. More studies focusing on diverse and large cohorts are needed to represent population variability, as most studies currently emphasize Western populations. Longitudinal multi-omics studies could provide insight into the complex progression of MDD, the illness, but such data sets are lacking [106]. A few of the ethical issues involved are the privacy of the data and the fair distribution of access to precision therapies.

The integration of proteomics, metabolomics, and epigenomics is more than just an approach to multi-omics; it redefines therapeutics in this example, demonstrating that multi-omics integration is revolutionizing MDD research by unraveling its underlying molecular complexity. Combining diverse biological data enables the discovery of biomarkers, patient stratification, and the development of targeted interventions. Continued advancements in computational methods and cohort diversity will further unlock its potential, paving the way for precision psychiatry.

## Novel Drug Targets Identified Through Multi-Omics

Multi-omics integration has uncovered new dysregulated pathways in MDD beyond monoamine (serotonin/norepinephrine) signaling, including: (1) neuroinflammation, evidenced by increased pro-inflammatory cytokines and activated microglia; (2) decreased synaptic plasticity, which comes from problems with BDNF-TrkB signaling; (3) mitochondrial metabolism, particularly related to altered kynurenine pathway activity; and (4) stress-induced gene expression changes, such as altered DNA methylation states (Table 3). Multi-omics techniques, which encompass proteomics, transcriptomics, genomics, epigenomics, and metabolomics, have systematically identified key pathways and molecules as targets for drug development, ushering in an era of precision medicine [107]. This updated review synthesizes important findings rather than reiterating each one.

**Table 3:** **Novel drug targets identified through multi-omics**. Candidate therapeutic targets for MDD, with supporting omics evidence, activity data, and development stage.

Target System	Key Molecular Targets	Mechanism	Potential Therapeutic Strategies	Ref.
**Glutamatergic System**	NMDA receptor (GRIN2B), mGlu5 receptor	Dysregulation of glutamate transmission, synaptic plasticity impairment	NMDA antagonists (e.g., ketamine), mGlu5 modulators	[113]
**Immune-Inflammatory Pathways**	IL-1RA, CRP, CCL2, NF-κB signaling	Chronic inflammation, elevated pro-inflammatory cytokines, neuroinflammation	Anti-inflammatory drugs, NF-κB inhibitors, cytokine modulators	[114]
**Gut-Brain Axis**	Microbial metabolites (e.g., short-chain fatty acids)	Dysbiosis, gut-brain signaling disruption, altered neurotransmitter synthesis	Probiotics, prebiotics, fecal microbiota transplantation (FMT)	[115]
**Neuroplasticity and Synaptic Regulation**	BDNF, SAMD3, NEGR1, mTORC1	Impaired synaptic connectivity, reduced neurogenesis, altered plasticity	BDNF mimetics, mTORC1 activators (e.g., NV-5138), neurotrophic agents	[116]
**Opioidergic System**	κ-Opioid receptor (KOR)	Dysregulation of stress response, altered reward processing	κ-receptor antagonists (e.g., JDTic), opioid modulators	[117]
**Monoaminergic System**	SERT (SLC6A4), DRD2	Reduced monoamine availability, impaired neurotransmission	SSRIs, SNRIs, atypical antipsychotics	[118]
**Endocannabinoid System**	CB1, CB2 receptors	Dysregulation of stress and emotional processing	CB1/CB2 modulators, FAAH inhibitors	[119]
**Circadian Rhythm Regulators**	CLOCK, BMAL1	Disrupted sleep-wake cycles, altered circadian gene expression	Melatonin receptor agonists, circadian rhythm stabilizers	[120]

Importantly, utilizing parameters such as proteomics of plasma as well as transcriptomics of postmortem MDD subjects, have consistently demonstrated that individuals with treatment-resistant MDD have elevated plasma levels of interleukin-6 (IL-6) [108]. In addition, transcriptomic analyses corroborated this finding based on increased IL-6 receptor (IL-6R) expression in the dorsolateral prefrontal cortex from postmortem brain tissue [109]. Indeed, IL-6 signaling is a prominent inflammatory pathway in the pathophysiology of MDD in individuals with higher baseline inflammatory markers [110]. Ultimately, a Phase II clinical trial of the drug tocilizumab (an IL-6R antagonist) given to treatment-resistant MDD patients demonstrated notable improvements in anhedonia, thereby establishing IL-6 as a suitable therapeutic target [111]. The multitude of clinical and translational model work informs the bio-signaling mechanistic underpinnings and strongly implies, providing strong evidence to support multi-omics analytical techniques in isolating biologically relevant and actionable molecules from endogenous inflammatory cascades [112].

Furthermore, genomic and epigenomic analyses, such as genome-wide association analyses, have identified the FK506 binding protein 5 (*FKBP5*) gene, specifically the rs1360780 variant, as involved in glucocorticoid resistance, a characteristic of MDD [121]. Preclinical interventions with SAFit2, an FKBP5 ligand, enhanced stress resilience in primate models, suggesting that FKBP5 may be a potential target for augmenting stress-coping processes [36]. The clinical and translational implications of FKBP5 dysregulation, considering the potential of FKBP5 inhibition as a therapeutic agent in stress-related diseases [121]. The FK506 binding protein 51 (FKBP5) is a repressor of the glucocorticoid receptor (GR), the primary regulator of HPA axis function, and FKBP5 polymorphisms have been consistently associated with stress-related disorders in humans [122]. It also involves the design of selective FKBP5 inhibitors and their potential therapeutic applications. Metabolic dysregulation presents another treatment strategy. Metabolomic analysis revealed disturbances in the kynurenine-to-tryptophan ratio, which differentiates MDD subtypes and associates metabolic dysregulation with clinical heterogeneity [123]. Clinical trials, including those testing the IDO1 inhibitor epacadostat (NCT04202627), are investigating these metabolic targets, particularly for inflammatory MDD subtypes, highlighting the clinical relevance of metabolomics in personalized treatment [124].

Epigenetic processes and glial cell function further amplify the therapeutic potential. Epigenome-wide association studies have linked histone deacetylase (HDAC) overexpression with childhood trauma and MDD susceptibility, shaping HDACs as a molecular interface between early stress and depression [10]. HDAC inhibitors, such as vorinostat, are predicted to be studied in relation to stress-related subtypes of MDD [125].

Single-cell transcriptomics and proteomics have also revealed microglial activation, with the TREM2/SYK signaling pathway identified in a recent study as a primary mediator of neuroinflammation [126]. Blocking SYK using drugs such as fostamatinib offers a hopeful approach to counteracting neuroinflammation and reducing depression symptoms [115]. Certain new receptor-based targets highlight that glutamate dysregulation is a hallmark of MDD, and multi-omics studies have emphasized the pivotal role of NMDA (especially GRIN2B-encoded GluN2B subunits) and mGlu5 receptors in the synaptic plasticity impairments that have been found in MDD pathophysiology [127]. Proteomic and metabolomic data also suggest the glutamatergic system as a primary driver for rapid-acting antidepressants, particularly ketamine's NMDA receptor modulation [127].

At the same time, immune dysregulation is characterized by increased inflammatory markers (IL-1RA, CRP, CCL2) and NF-κB pathway activation characterizes a clinically distinct subgroup of MDD, and anti-inflammatory interventions become personalized therapeutic strategies [128]. The gut-brain axis is also involved in causing MDD via microbial dysbiosis, with metabolomic profiling showing dysregulated short-chain fatty acid content and microbial diversity, providing probiotics and microbial metabolite modulation as new therapeutic options [129].

Moreover, multi-omics methodologies have implicated disrupted neuroplasticity pathways, such as BDNF signaling and synaptic gene regulation (e.g., SAMD3, NEGR1), with mTORC1 activators as possible synaptic enhancers. Lastly, the opioid system, specifically κ-receptor upregulation, is a stress-associated MDD mechanism, with κ-antagonists being a therapeutic option for treatment-resistant depression [130]. Overall, these multi-omics-derived targets, glutamatergic, immune, microbial, neuroplasticity, and opioid pathways highlight the heterogeneity of MDD and present an avenue for mechanistically targeted, rapid-onset therapeutics.

## Novel drugs in MDD using structure-based approaches

MDD is a complex psychiatric condition, and current treatments often have limited efficacy and significant side effects. Structural biology approaches, including X-ray crystallography, cryo-EM, and NMR, combined with docking, molecular dynamics (MD) simulations, are playing an increasingly important role in drug discovery for MDD. These techniques help in target identification, binding site characterization, and rational drug design. Here are some novel drugs in clinical trials for MDD that have benefited from structural biology and computational approaches (Table 4).

**Table 4:** **Representative clinical trials in MDD**. Selected ongoing and completed clinical trials of emerging therapies for major depressive disorder.

Drug Name	Mechanism of Action	Clinical Trial Stage	Ref.
**Psilocybin**	5-HT2A receptor agonist (rapid synaptic plasticity)	Phase III (COMPASS Pathways: NCT03775200)	[131]
**Esketamine (Spravato)**	NMDA receptor antagonist (glutamate modulation)	Approved (2019), Phase IV post-marketing studies	[132]
**REL-1017 (Esmethadone)**	NMDA receptor antagonist (non-competitive)	Phase III (NCT04688164)	[133]
**AXS-05 (Dextromethorphan-Bupropion)**	NMDA antagonist + σ1 agonist + norepinephrine-dopamine reuptake inhibition	Phase III (NCT04163094)	[134]
**Zuranolone (SAGE-217)**	Positive allosteric modulator of GABA<sub>A</sub> receptors (neurosteroids)	Approved (2023) for postpartum depression; Phase III for MDD (NCT04476030)	[135]
**Rupertixibat (LY-3522347)**	Kappa opioid receptor (KOR) antagonist	Phase II (NCT04247542	[136]
**BI 1358894**	TRPC4/5 ion channel blocker (amygdala hyperactivity modulation)	Phase II (NCT04776688)	[137]
**Ansofaxine (LY-03005)**	Triple reuptake inhibitor (SERT, NET, DAT)	Phase III (NCT05236868)	[138]
**NRX-101 (D-cycloserine + Lurasidone)**	NMDA + D2/5-HT2A modulation (suicidal MDD)	Phase IIb (NCT04018542)	[139]
**MIJ821 (OnabotulinumtoxinA)**	Synaptic vesicle protein modulation (rapid antidepressant)	Phase II (NCT04272138)	[140]

## NMDA receptor modulators (targeting glutamatergic system)

High-resolution structural biology, utilizing X-ray crystallography and cryo-electron microscopy, has played a crucial role in the rational design of NMDA receptor modulators, exemplified by the development of Rapastinel (GLYX-13). Structural insights from complexes like GluN1-GluN2B elucidated the conformational landscape of the glycine-binding site on the GluN1 subunit, revealing key interactions that guided the optimization of Rapastinel for enhanced binding affinity while maintaining partial agonism [141]. Although Phase III clinical trials for Rapastinel were ultimately discontinued, its development highlights the utility of integrating molecular dynamics simulations with crystallographic data to engineer next-generation glycine-site modulators with improved pharmacological profiles [142]. Future efforts in this area can leverage recent cryo-EM structures of full-length NMDA receptors (e.g.PDB ID: 7EU8) to achieve more refined subunit-specific targeting, potentially overcoming the limitations encountered with earlier compounds like Rapastinel [143] (Fig. 2A).

The rational design of the prodrug AV-101 (L-4-chlorokynurenine), which metabolizes to the active glycine-site NMDA receptor antagonist 7-chlorokynurenic acid (7-Cl-KYNA) was significantly facilitated by structural elucidation of the NMDA receptor's ligand-binding domain [144]. High-resolution structural techniques, including X-ray crystallography and cryo-electron microscopy (cryo-EM) of GluN1-GluN2B receptor complexes bound to glycine-site antagonists (e.g., PDB IDs 4PE5, 5I57), unveiled crucial interactions governing antagonist binding to the GluN1 subunit. These structural insights informed the strategic optimization of 7-Cl-KYNA to achieve enhanced binding affinity and reduced off-target activity [145]. Complementary computational approaches, such as molecular docking and dynamics simulations, played a vital role in predicting the binding mode and stability of 7-Cl-KYNA within the glycine-binding pocket, further refining its pharmacological characteristics. This synergistic application of computational modeling and experimental structural biology has been pivotal in the development of AV-101 as a promising rapid-acting antidepressant, currently undergoing Phase II clinical evaluation [146]. Future endeavors to improve subunit selectivity and therapeutic efficacy may capitalize on recent cryo-EM structures of the complete NMDA receptor (e.g., PDB ID: 7EU8), offering a more comprehensive understanding of ligand interactions within the intact receptor architecture [147].

**Figure 2.**
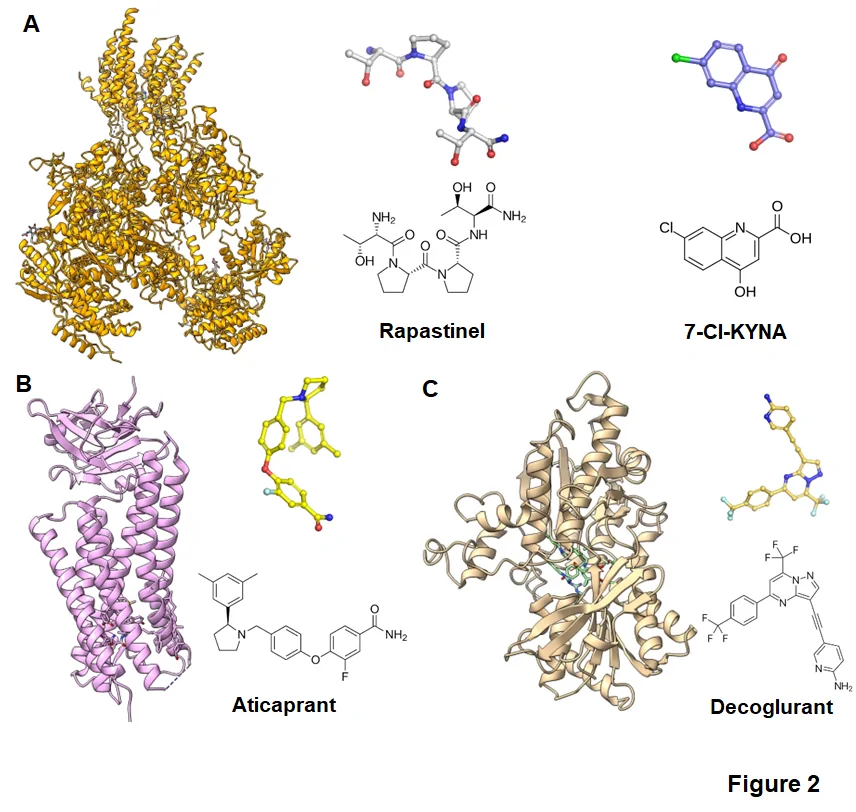
**Structure-guided targets and compounds relevant to MDD**. The left panels display cartoon ribbon structures of the NMDA receptor, the Kappa Opioid Receptor (KOR), and mGluR2/3, along with their respective modulators. (**A**) Three-dimensional cartoon representation of the NMDA receptor, illustrating its tetrameric architecture and key binding sites. The right panel shows molecular structures of Rapastinel, a positive allosteric modulator of the NMDA receptor, and 7-chlorokynurenic acid (7-Cl-KYNA), a competitive NMDA receptor antagonist. (**B**) Molecular architecture of the KOR and its antagonist J-NJ-67953964, highlighting key binding interactions and structural features critical for its antagonistic activity. (**C**) Three-dimensional structure of mGluR2/3 (PDB ID: 4XAS), illustrating the binding site of Decoglurant (RG1578) (RG1578). Structural insights into Decoglurant as a negative allosteric modulator of mGluR2/3 with implications for novel therapeutic strategies in major depressive disorder (MDD). The structures shown were selected for three reasons: (1) biological relevance, the protein/receptor has strong multi-omics support linking it to MDD (genetic, transcriptomic, proteomic or metabolomic signals); (2) therapeutic interest, small molecules or biologics targeting this structure have entered clinical development for depressive disorders; and (3) structural data availability, a high-resolution structure (X-ray/cryo-EM/NMR) or reliable homology model exists and has guided ligand optimization.

## Opioid receptor kappa (KOR) antagonists

High-resolution structural biology techniques, specifically cryo-electron microscopy and X-ray crystallography, have been instrumental in the rational design of the selective kappa opioid receptor (KOR) antagonist Aticaprant (LY-2456302/CERC-501) [148]. Cryo-EM studies, exemplified by the structure of KOR in complex with the antagonist JDTic (PDB ID: 6B73), have elucidated key interactions within the orthosteric binding pocket involving residues Asp138, Tyr139, and Tyr312 (Fig. 2B). These structural insights are crucial for understanding antagonist selectivity against other opioid receptor subtypes, such as MOR and DOR [149]. Leveraging this structural information, Aticaprant was optimized for enhanced KOR affinity and reduced off-target binding. Complementary molecular dynamics simulations have provided dynamic perspectives on the conformational changes induced upon antagonist binding, aiding in the development of compounds with favorable pharmacokinetic properties. Considering the involvement of KOR in stress-related dysphoria, this structure-guided drug design strategy offers a promising avenue for the creation of novel antidepressants characterized by rapid and sustained therapeutic effects.

## mGluR2/3 negative allosteric modulators

The rational design of Decoglurant (RG1578), a negative allosteric modulator of mGluR2/3 (PDB IDs: 4XAS; 4XAR), exemplifies the application of structural biology in drug discovery (Fig. 2C). High-resolution X-ray structures of related metabotropic glutamate receptor transmembrane domains (mGluR1 and mGluR5) served as crucial templates for homology modeling mGluR2/3, owing to the conserved architecture of their allosteric binding sites [150]. These structural insights unveiled key interactions between specific transmembrane helices (TM3, TM5, and TM7) and allosteric modulators, enabling the targeted design of Decoglurant to selectively attenuate glutamate signaling without direct competition at the orthosteric site [151]. Despite its discontinuation in Phase II clinical trials, the structural optimization of Decoglurant underscores the potential of cryo-electron microscopy advancements, which have since provided direct structural information for mGluR2, to refine the development of next-generation mGluR2/3 modulators further. Future efforts can leverage molecular modelling to optimize binding kinetics and functional modulation, thereby overcoming the limitations encountered with earlier compounds and potentially yielding more efficacious and selective therapeutics [152].

## 5-HT2A receptor inverse agonists (psychedelic derivatives)

Cryo-electron microscopy has recently yielded high-resolution structures of the 5-HT2A serotonin receptor bound to psychedelic compounds (PDB-8ZMG)-, including psilocin and LSD, revealing critical binding interactions and the stabilization of an active receptor conformation. These structural insights highlight a specific aromatic cluster within the orthosteric binding pocket that is implicated in their hallucinogenic properties [153]. Leveraging this knowledge, current research efforts are focused on structure-guided design strategies, employing mutagenesis and molecular dynamics simulations to identify key residues that decouple psychedelic effects from potential antidepressant efficacy [154], as exemplified by the ongoing development of non-hallucinogenic analogs, such as Psilocybin (Fig. 3A).

Furthermore, the structures of 5-HT2A in complex with biased agonists and inverse agonists provide a framework for the rational design of next-generation therapeutics with enhanced safety profiles. The detailed understanding of 5-HT2A-psilocin interactions through structural biology marks a significant advancement in the field, paving the way for the refinement of psilocybin-like compounds for broader clinical applications. Future directions emphasize the exploration of allosteric modulation and biased signaling guided by high-resolution dynamic studies [155].

Psychedelic-assisted therapies (e.g., psilocybin, LSD, and related 5-HT2A-acting compounds) have re-emerged as promising class for MDD and TRD. Early randomized and open-label trials show rapid, sometimes durable, symptom reduction with single-session or limited-session administration when combined with psychotherapy. Mechanistic hypotheses include transient network desegregation and enhance neural plasticity that may permit therapeutic relearning. However, the evidence base is currently dominated by small trials, with variable blinding and limited long-term safety data. Ongoing Phase II/III trials will be critical to defining efficacy, risk-benefit, and optimal patient selection. Structure-guided work on 5-HT2A and biased agonists is now attempting to dissociate hallucinogenic and therapeutic signaling [156].

## Sigma-1 receptor agonists

High-resolution X-ray crystallography of the sigma-1 receptor (S1R) ligand-binding domain (PDB ID: 6DJZ) [157] has provided a structural basis for understanding the agonistic activity of Ansofaxine (LY03005), a serotonin-norepinephrine-dopamine reuptake inhibitor with S1R affinity (Fig. 3B). These structural studies revealed a versatile binding pocket within S1R, capable of accommodating diverse ligands, including the known agonist (+)-pentazocine. Utilizing these insights, the binding mode of Ansofaxine has been refined, highlighting crucial interactions with residues such as Glu172 and Asp126, which are critical for stabilizing the receptor conformations associated with agonist activity.

Complementary molecular dynamics simulations further suggest that S1R agonists, including Ansofaxine, enhance chaperone-like function by influencing endoplasmic reticulum membrane dynamics, potentially contributing to the observed neuroprotective and antidepressant effects [158]. Considering S1R's involvement in neuroplasticity and oxidative stress reduction, the dual action of Ansofaxine monoamine reuptake inhibition coupled with S1R activation presents a compelling approach for treatment-resistant depression. Future structure-based drug design strategies, potentially informed by cryo-EM structures of S1R in lipid environments (e.g., PDB ID: 6DK1), could facilitate the development of optimized analogs with improved brain penetration and selectivity profiles, minimizing off-target interactions, such as those with opioid receptors.

**Figure 3.**
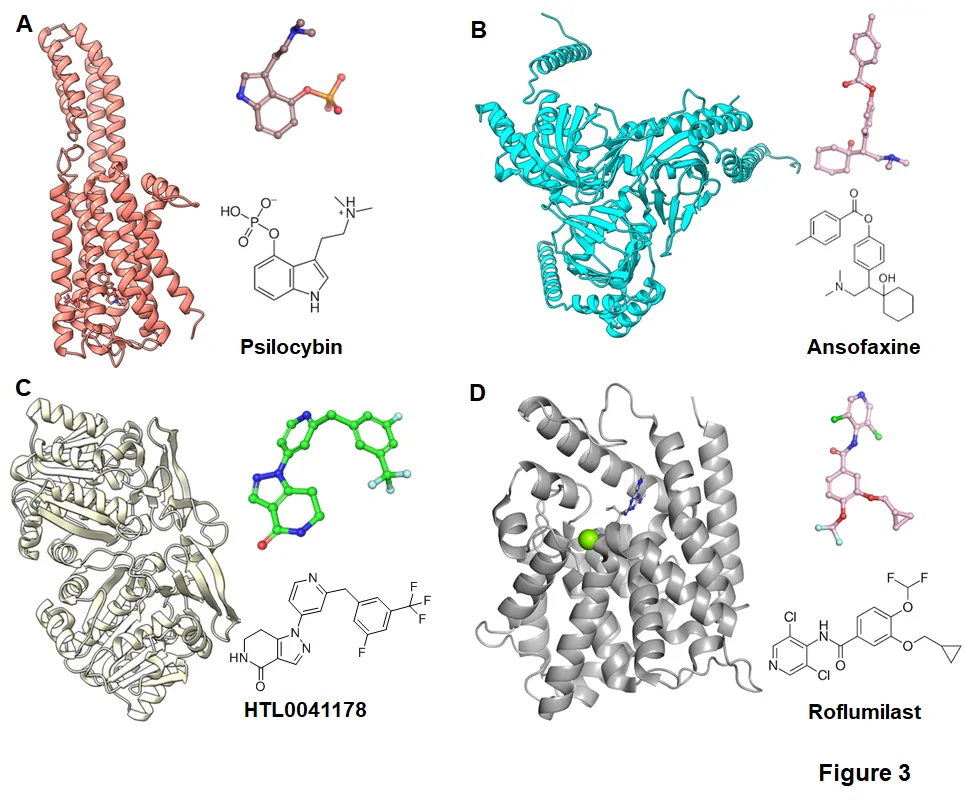
**Additional targets and their structural insights into 5-HT2A Serotonin receptor, Sigma-1 Receptor (S1R), GPR52, and PDE4**. (**A**) Three-dimensional structure of the 5-HT2A serotonin receptor (PDB ID: 8ZMG) bound to psychedelic compound COMP360. (**B**) Crystal structure of the Sigma-1 Receptor (S1R) ligand-binding domain (PDB ID: 6DJZ) and its agonist Ansofaxine (LY03005). (**C**) Three-dimensional 3D structure of GPR52 (PDB ID: 7XIG), an orphan G protein-coupled receptor (and its agonist HTL0041178) implicated in dopaminergic signaling pathways relevant to MDD. (**D**) Three-dimensional structure of phosphodiesterase-4 (PDE4), derived from crystallographic data and its inhibitor Roflumilast.

## GPR52 agonists (novel GPCR target)

GPR52, an orphan G protein-coupled receptor implicated in dopaminergic signaling relevant to MDD, represents a compelling therapeutic target. High-resolution structural information obtained through cryo-electron microscopy (e.g., PDB ID: 7XIG) has unveiled critical features of its ligand-binding sites, enabling structure-based drug design efforts to identify selective agonists, such as HTL0041178 [159] (Fig. 3C). These agonists, engineered for optimized affinity and specificity, have been further refined through molecular dynamics simulations that elucidate receptor conformational changes upon binding and aid in developing candidates with enhanced pharmacokinetic properties, including improved blood-brain barrier permeability and metabolic stability.

The preferential expression of GPR52 within brain regions rich in dopamine suggests that these agonists could offer a targeted approach to modulate depressive symptoms, potentially minimizing the off-target effects associated with traditional monoaminergic antidepressants. Future research directions should focus on exploring biased agonism at GPR52 to selectively engage downstream signaling pathways, such as Gαs or β-arrestin recruitment, thereby enhancing the precision of therapeutic interventions for MDD [160].

## PDE4 inhibitors (targeting neuroinflammation)

The high-resolution 3D structure has played a crucial role in exploring the potential of a phosphodiesterase-4 (PDE4) repurposing inhibitor, roflumilast, initially indicated for COPD, as a therapeutic for MDD (Fig. 3D). X-ray crystallographic analyses of PDE4B bound to roflumilast have elucidated key interactions within the enzyme's catalytic domain, specifically highlighting the stabilization of the glutamine-switch and engagement of the hydrophobic pocket [161]. These structural details have informed strategies to optimize the selectivity and potency of PDE4 inhibitors, aiming to minimize interactions with other PDE isoforms, such as PDE4D, known to be associated with emetic side effects. Complementary molecular dynamics simulations have provided a dynamic understanding of the active site's conformational landscape.

Considering the involvement of neuroinflammation in MDD, these structural insights into PDE4-inhibitor interactions provide a molecular basis for investigating roflumilast's anti-inflammatory and neuroprotective properties in the context of this psychiatric disorder [162]. Future endeavors in developing advanced PDE4 modulators could leverage cryo-electron microscopy structures of complete PDE4 complexes further to improve drug penetration into the central nervous system while mitigating undesirable effects.

## Emerging therapies

Recent advances in neuroscience and technology have ushered in a new era of innovative therapies for MDD, targeting novel mechanisms to address treatment-resistant cases and improve patient outcomes. Rapid-acting antidepressants, such as ketamine and its FDA-approved derivative esketamine (Spravato), act by antagonizing the NMDA receptor to enhance synaptic plasticity through increased expression of brain-derived neurotrophic factor (BDNF), providing symptom relief within hours to days [136].

Psychedelics, including psilocybin, LSD, and DMT/ayahuasca, target 5-HT2A receptors to promote neuroplasticity and reset maladaptive neural circuits, with psilocybin advancing to Phase III trials and anticipated regulatory review around 2025-2026 [163]. Anti-inflammatory approaches, including minocycline and TNF-α inhibitors such as infliximab, target MDD with elevated inflammation (e.g., high CRP levels), complemented by the anti-inflammatory effects of psilocybin. Metabolic interventions, such as the ketogenic diet, modulate GABA/glutamate balance, while psychobiotics (e.g., Lactobacillus, Bifidobacterium) and experimental fecal microbiota transplants explore the gut-brain axis [164].

Stem-cell-based approaches are an emerging, preclinical-early clinical avenue for MDD that operate through neurotrophic, anti-inflammatory, and circuit-repair mechanisms. Preclinical studies suggest that mesenchymal stromal cells (MSCs) and neural stem/progenitor cells can enhance hippocampal neurogenesis, modulate microglial activation, and increase the expression of neurotrophic factors. Although early-phase human studies are limited, they provide proof of concept for the safety and biological activity of these cells. Current limitations include delivery, cell-type selection, immune compatibility, and scalability; thus, stem-cell strategies are best viewed as complementary to small-molecule and neuromodulation approaches, and warrant controlled early-phase trials with rigorous biomarker endpoints [165].

Digital therapeutics, like the FDA-cleared Rejoyn app, combine cognitive training with emotional regulation, and AI-driven tools leverage genetics and symptom profiles to personalize treatment. Emerging targets, such as kappa-opioid antagonists (e.g., BTRX-246040) and neurosteroids like zuranolone (FDA-approved for postpartum depression), further diversify the therapeutic landscape [128]. Despite challenges in accessibility, long-term efficacy, and integration into clinical practice, these therapies highlight a shift toward rapid-acting, neuroplasticity-enhancing, and personalized approaches, offering hope for patients with previously intractable MDD.

## Challenges and future directions

Multi-omics approaches face several challenges despite their promise. High-dimensional data necessitate robust computational frameworks to prevent overfitting, especially when working with small sample sizes. Variability in MDD descriptions and treatment protocols across studies complicates the interpretation of meta-analyses. Additionally, translating omics findings into clinical practice remains challenging due to the need for cost-effective, non-invasive biomarkers. Future research in multi-omics for MDD should prioritize large-scale, multi-site studies encompassing diverse populations to improve the generalizability of findings. Longitudinal designs are crucial for tracking dynamic omics alterations and identifying biomarkers that evolve. Advanced analytical approaches, including machine learning and artificial intelligence, are necessary to integrate complex multi-omics datasets and accurately predict individual treatment responses. Translating these discoveries into clinical practice requires the development of accessible point-of-care omics assays for real-time treatment stratification. Finally, investigating the gut-brain axis and exploring microbial-based therapies offers a promising avenue to complement traditional pharmacotherapies for MDD.

## Conclusions

Integrative multi-omics has transformed understanding of MDD from symptom-based descriptions toward molecularly stratified subtypes characterized by convergent dysregulation in synaptic plasticity, immune-inflammatory signaling, HPA-axis regulation, energy metabolism and gut-brain interactions. When coupled with AI-driven network modeling and structure-based drug design, these data provide a roadmap for biomarker-guided trials and mechanism-based therapeutics, e.g., rapid-acting glutamatergic agents, kappa-opioid antagonists, anti-inflammatory interventions, psychedelics and microbiome-based strategies. However, key translational hurdles remain. First, replication and generalizability are limited by cohort heterogeneity and under-representation of non-European ancestries. Second, high-dimensional omics data require standardized pre-analytic pipelines and harmonized metadata to avoid batch artifacts and false positives. Third, moving biomarkers into clinical practice demands cost-effective, minimally invasive assays and prospective validation in interventional trials. Finally, the safety and durability of many emerging therapies, for example, psychedelics and cell-based approaches, require longer follow-up and larger controlled trials. To accelerate translation, we recommend: (1) large, multi-ethnic, longitudinal multi-site studies with harmonized protocols; (2) prospective biomarker-driven trial designs, i.e., biomarker-stratified/biomarker-adaptive trials; (3) open sharing of raw multi-omics data and analytic code; and (4) cross-disciplinary consortia linking omics, structural biology, and clinical trial teams to convert molecular insights into optimized therapeutics rapidly. With these steps, multi-omics-informed precision psychiatry can move from promise to practice for people living with MDD.
